# Ultrasound-Guided Radiofrequency Ablation *Versus* Surgical Resection for the Treatment of T1bN0M0 Papillary Thyroid Carcinoma in Different Age Groups

**DOI:** 10.3389/fendo.2021.734432

**Published:** 2021-08-27

**Authors:** Hongying He, Rilige Wu, Jiahang Zhao, Qing Song, Yan Zhang, Yukun Luo

**Affiliations:** ^1^School of Medicine, Nankai University, Tianjin, China; ^2^Department of Ultrasound, The First Medical Center of Chinese People’s Liberation Army (PLA) General Hospital, Beijing, China; ^3^Medical Big Data Research Center, The First Medical Center of Chinese PLA General Hospital, Beijing, China

**Keywords:** papillary thyroid carcinoma, radiofrequency ablation, surgery, disease-free survival, complication

## Abstract

**Purpose:**

We aimed to compare the efficacy and safety of radiofrequency ablation (RFA) to that of surgical resection (SR) in patients with T1bN0M0 papillary thyroid carcinoma (PTC) in different age groups.

**Methods:**

Totally, 204 patients with an isolated, solitary, intrathyroidal T1bN0M0 PTC, who underwent either RFA (n=94) or SR (n=110) between April 2014 and December 2019, were retrospectively enrolled and were divided into two subgroups according to age (<45 years, ≧45 years). Patients with pathologically aggressive or advanced lesions were excluded from the study. Tumor progression and procedural complications were the primary and secondary endpoints, respectively. Tumor recurrence *in situ*, newly discovered tumors, lymph node involvement, or distant metastases indicated tumor progression. Complications included pain, fever, voice change, choking, numbness in the limbs, and cardiac events. Incidence rates of all endpoint events were compared between different age subgroups.

**Results:**

There were no significant differences in age, sex, and tumor size between the treatment groups. While the RFA group incurred less cost and experienced significantly shorter operative duration than the SR group, no significant differences were observed in incidences of both tumor progression and complications. Further, subgroup analysis of patients <45 years *versus* those ≧45 years showed no significant differences in the incidence of tumor progression and complications within or between different treatment groups. Older patients in the SR group incurred higher hospital costs than younger counterparts, but this difference was not observed in the RFA group.

**Conclusions:**

Our results indicated that RFA had a similar prognosis as that of SR but was associated with lower overall cost in both young (<45 years) and middle-aged patients (≧45 years) with T1bN0M0 PTC. Therefore, RFA may be an effective and safe alternative to surgery for the treatment of patients with T1bN0M0 PTC.

## Introduction

Papillary thyroid carcinoma (PTC) is the most common histological type of thyroid carcinoma, accounting for 70–90% of differentiated thyroid cancers (1, 2). Although the local recurrence rate of PTC can reach as high as 30%, the associated mortality rate is extremely low, with a 10-year overall survival rate >90% (2), due to its relatively mild biological behavior. The American Joint Committee on Cancer (AJCC) on the tumor, lymph node, metastasis (TNM) staging system defined T1bN0M0 PTC as that limited to the thyroid with tumor >1 cm but ≦2 cm in the greatest dimension with no evidence of locoregical lymph node involvement or of distant metastasis (3). Currently, the 2015 American Thyroid Association (ATA) guidelines (4) still recommend thyroid lobectomy as the first-line treatment for PTC. However, associated incidence of operative complications such as temporary or permanent recurrent laryngeal nerve paralysis, hypothyroidism, and hypoparathyroidism can range from 16.3 to 72.5%, according to previous studies (5–10).

Presently, overdiagnosis and overtreatment of PTC is one of the most controversial issues in the management of thyroid carcinoma (11, 12). Long-term active surveillance (AS) of patients with T1aN0M0 PTC performed in several institutions has revealed that the follow-up approach may constitute an appropriate, conservative management strategy (13–15). The 2015 ATA Management Guidelines for Adult Patients with Thyroid Nodules and Differentiated Thyroid Cancer also mentioned that AS may be employed as an alternative strategy in selected PTC patients (4). However, long-term follow-up on studies on AS for T1bN0M0 PTC have been rarely reported.

In comparison to active surveillance (AS) and surgical resection (SR), the treatment modality of radiofrequency ablation (RFA) utilizes frictional heating, which may lead to cell injury (16) in a bid to achieve E0 resection. RFA is characterized by its technical ease and non-invasive nature. To date, several leading institutes have reported successful outcomes with the use of RFA in the treatment of T1N0M0 PTC, recurrent thyroid cancers, and lymph node metastatic tumors (17–22). As compared to SR, RFA is associated with comparable long-term outcomes, shorter hospital stay, and fewer temporary and permanent complications (23, 24). Unlike AS, RFA allows safe and effective removal of tumors, thereby minimizing harmful consequences of potential growth of the malignancy. However, while tumor size and patient age are related to the prognosis of differentiated thyroid cancers (25, 26), there is a lack of long-term data on researches on the outcomes of RFA performed for the treatment of T1bN0M0 PTC across different age groups. In the present study, we aimed to compare the efficacy and safety of RFA with that of SR for T1bN0M0 PTC, in patients of different age groups.

## Material and Methods

This retrospective study was approved by the Institutional Review Board of the First Medical Center of the Chinese PLA General Hospital. We reviewed medical records of patients with T1bN0M0 PTC, who underwent RFA or surgery from April 2014 to December 2019. All patients provided written informed consent prior to a treatment procedure.

### Patients

**Figure 1:**
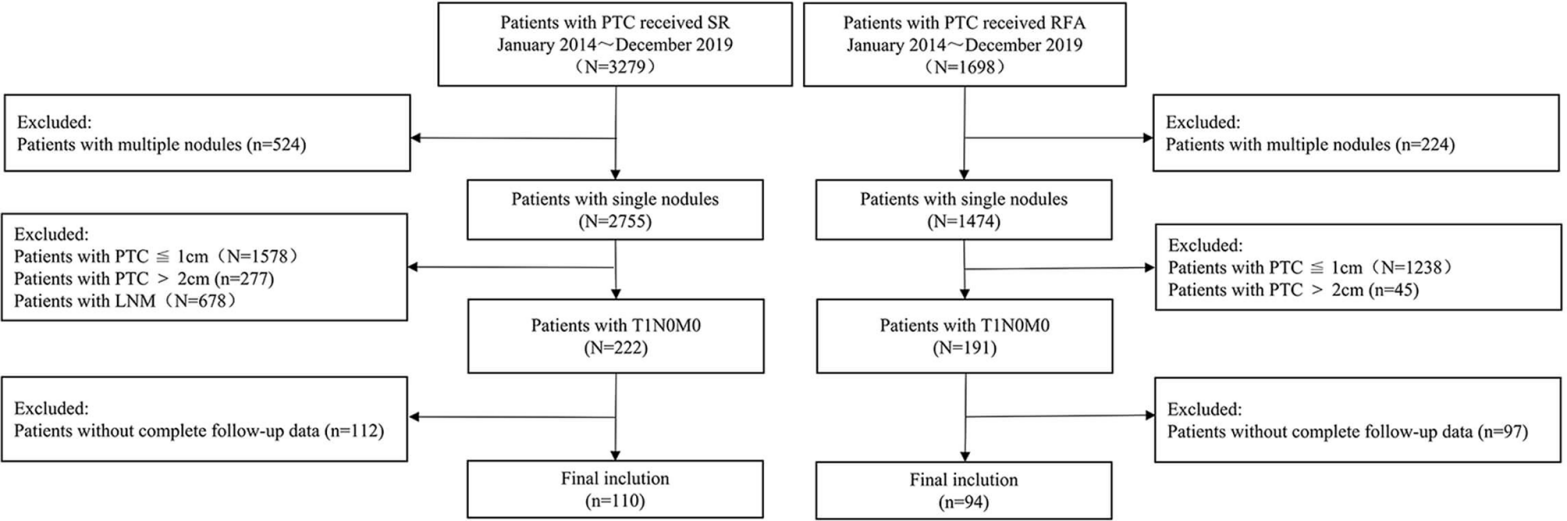
Flowchart of patient enrolment.

The eligibility criteria for inclusion in the RFA group were as follows: (1) cytologically confirmed PTC; (2) maximum tumor size between 1.0 and 2.0 cm on ultrasound; (3) no evidence of any extra-glandular invasion, lymph node metastasis, or distant metastasis; and (4) those who refused or were ineligible for surgical resection but desired reduction of the tumor burden. The exclusion criteria were the following: (1) age ≤18 years; (2) pregnant or breastfeeding women; (3) coagulation dysfunction; (4) PTC with aggressive histopathological subtypes (tall and columnar cells, diffuse follicular, and sclerosis); (5) severe cardiac pathology; and (6) incomplete follow-up data (**Figure 1**).

The enrolment criteria for inclusion in the open surgery group were (1) suspicious solitary thyroid nodule between 1.1 and 2.0 cm in diameter, detected on preoperative ultrasound examination; (2) no evidence of any extra-glandular invasion, lymph node metastasis, or distant metastasis; (3) confirmed PTC on histopathological examination of the resected tissue; and (4) complete follow-up data ≥12 months available.

### Pre-Ablation Evaluation

All patients underwent pre-ablation assessments, including imaging and laboratory tests. Ultrasound characteristics of the tumor including size (diameter in three dimensions), location (lobe distribution; upper or lower position), composition, echogenicity, margin (regular or irregular), aspect ratio (height/width), presence/absence of calcifications, and vascularity (divided into four levels according to the Adler blood flow grading) (27) were evaluated. Tumor volume was calculated as:

V=πabc/6

(20, 28), where *V* represents volume, and *a*, *b*, and *c* represent the tumor diameters in three dimensions. Contrast-enhanced ultrasonography (CEUS) was used to evaluate the tumor blood supply, capsular infiltration, and extra-glandular invasion. Diagnostic core needle biopsy (CNB) was performed for all patients in the RFA group to confirm the presence of thyroid malignancy. Laboratory tests mainly included thyroid function tests, blood coagulation profile, and complete blood count.

### Ablation Procedure

All biopsies and ablation procedures were performed using the Siemens Acuson Sequoia 512 device with a 6L3 linear array transducer, by an experienced ultrasound physician (YL, with ≥20 years of clinical experience in diagnosis and treatment of thyroid disease) to exclude possibility of bias induced either by the use of different machine systems or by different operators performing the ablation.

Patients were arranged in the supine position with the neck extended. Vital signs were continuously monitored during the procedure. Tumor blood supply and relationships among the recurrent laryngeal nerve, trachea, esophagus, and the PTC were carefully assessed to select the most suitable pathway for insertion of the needle or electrode. Local anesthetic (1% lidocaine hydrochloride) was injected into the subcutaneous tissue and in the anterior thyroid capsule using a 23-gauge needle. Normal saline was injected around the anterior thyroid capsule to prevent heat injury, in case the distance between the targeted ablation zone and surrounding critical structures in the neck was ≤5 mm, a step known as the “hydrodissection technique.”

After creating a safety margin, an electrode was inserted into the deepest part of the tumor, and the RF output was set at 3–6 W power. The moving-shot technique was used to achieve unit-by-unit ablation without generating excessive heat, which could cause a carbonization effect, adversely affecting the heat transfer. Adequate RFA was performed so that the area of ablation included an expanded zone involving at least 2 mm of the surrounding normal thyroid tissue, to radically eliminate tumor cells and prevent local recurrence. RFA was terminated when all the targeted areas were found to be hyperechoic on ultrasound. In order to prevent excessive ablation, CEUS was performed immediately after RFA to assess the microvasculature circulation dynamics and to determine the precise range of ablaton.^30–32^ All RFA data, including output power (Watt), duration (min), and total energy used (Kcal), were recorded.

### Surgical Procedure

SR was performed under general anesthesia. The decision to perform either total thyroidectomy or lobectomy was made in consensus by a team of experienced surgeons, and all patients underwent concurrent prophylactic central lymph node dissection.

### Follow-Up

Follow-up assessments were conducted at 1, 3, 6, and 12 months postoperatively and at every 6–12 months thereafter. In the RFA group, the ablation range was evaluated using VRR, which was calculated using the following formula:

VRR (%) = [(initial volume−final volume) ×100]/initial volume

Tumor progression in both groups was defined as tumor recurrence *in situ*, newly developed cancers, lymph node metastasis, or distant metastasis, as confirmed on biopsy. Any additional administered for tumor progression was also recorded. We collected data of complications that occurred both intraoperatively and during the follow-up period.

### Statistical Analysis

The SPSS software (version 24.0, IBM Corp.) was used for all statistical analyses. Chi-square or Fisher’s exact probability tests were used for the comparison of categorical variables, and Student’s t-test was used for the comparison of continuous variables. The VRR change was tested using the paired-sample Wilcoxon signed-rank test. The level of statistical significance was set at P < 0.05.

## Results

### Baseline Characteristics

Baseline clinical characteristics and outcomes are summarized in **Table 1**. A total of 204 patients were included in this study, of whom 94 (46.1%) and 110 (53.9%) underwent RFA and SR, respectively. There were no significant differences between both groups in terms of factors including mean age (P=0.924), sex (P=0.106), and tumor volume (P=0.814). However, some tumor futures, including aspect ratio (P<0.001), type of edge (P<0.001), and extent of calcification (P<0.001), differed significantly between the treatment groups. Patients aged <45 years and those of age ≧45 years each accounted for half of the participants if the RFA group. In the SR group, the former and the latter age-group patients accounted for 53.6 and 46.4% participants, respectively. There was no statistical difference in age distribution between both treatment groups (P=0.604).

**Table 1:** Baseline characteristics.

Variable		Total	RFA	SR	P
**Sex**	Female	164 (80.4%)	71 (75.50%)	93 (84.5%)	0.106
	Male	40 (19.6%)	23 (24.50%)	17 (15.5%)	
**Age**	Mean age	43.86	43.94	43.79	0.924
	≦45	106 (52%)	47 (50%)	59 (53.6%)	0.604
	>45	98 (48%)	47 (50%)	51 (46.4%)	
**Volume (cm^3^)**	Mean	0.815	0.795	0.833	0.814
**Aspect_ratio**	<1	80 (39.2%)	63 (67%)	17 (15.5%)	<0.001*
	>1	124 (60.8%)	31 (33%)	93 (84.5%)	
**Edge**	Yes	36 (17.6%)	31 (33%)	5 (4.5%)	<0.001*
	No	168 (82.4%)	63 (67%)	105 (95.5%)	
**Calcification**	No	47 (23%)	29 (30.9%)	18 (16.4%)	<0.001*
	<1 mm	107 (52.5%)	25 (26.6%)	82 (74.5%)	
	1 mm< & <2 mm	12 (5.9%)	5 (5.3%)	7 (6.4%)	
	>3 mm	38 (18.6%)	35 (37.2%)	3 (2.7%)	
**Location 1**	Left lobe	77 (37.7%)	33 (35.1%)	44 (40.0%)	0.377
	Right lobe	96 (47.1%)	49 (52.1%)	47 (42.7%)	
	Isthmus	31 (15.2%)	12 (12.8%)	19 (17.3%)	
**Location 2**	Upper	36 (25.9%)	19 (23.2%)	17 (29.8%)	0.368
	Mid	56 (40.3%)	37 (45.1%)	19 (33.3%)	
	Lower	47 (33.8%)	26 (31.7%)	21 (36.8%)	
**CDFI**	0	46 (22.5%)	25 (26.6%)	21 (19.1%)	0.455
	I	61 (29.9%)	29 (30.9%)	32 (29.1%)	
	II	57 (27.9%)	25 (26.6%)	32 (29.1%)	
	III	40 (19.6%)	15 (16%)	25 (22.7%)	

RFA, radiofrequency ablation; SR, surgical resection; CDFI, color Doppler flow imaging. *P < 0.05.

### Characteristics and Outcome of RFA According to Age Subgroups

There were no significant differences in demographic features and tumor characteristics across both age subgroups (**Table 2**). In addition, RFA parameters including power of output (P=0.739), procedure duration (P=0.450), total energy consumed (P=0.736), and hospital costs (P=0.537) showed no differences between patients aged <45 years and those ≧45 years of age. Complications occurred in 2 (2.0%) patients, one whom was <45 years old (moderate fever) and the other was ≧45 years of age (hoarseness). As of April 2021, while 4 (4.3%) patients developed tumor progression, no significant difference was observed between the defined age subgroups.

**Table 2:** Characteristics and outcomes of RFA according to age subgroups.

RFA		Total	<45	≧45	p
Sex	Female	71 (75.5%)	37 (78.7%)	34 (72.3%)	0.632
	Male	23 (24.5%)	10 (21.3%)	13 (27.7%)	
Aspect_ratio	<1	63 (67%)	30 (63.8%)	33 (70.2%)	0.510
	>1	31 (33%)	17 (36.2%)	14 (29.8%)	
Edge	Yes	31 (33%)	19 (40.4%)	12 (25.5%)	0.125
	No	63 (67%)	28 (59.6%)	35 (74.5%)	
CDFI	0	25 (26.6%)	13 (27.7%)	12 (25.5%)	0.928
	I	29 (30.9%)	13 (27.7%)	16 (34.0%)	
	II	25 (26.6%)	13 (27.7%)	12 (25.5%)	
	III	15 (16%)	8 (17%)	7 (14.9%)	
Calcification	No	29 (30.9%)	16 (34.0%)	13 (27.7%)	0.536
	<1 mm	25 (26.6%)	13 (27.7%)	12 (25.5%)	
	1 mm< & <2 mm	5 (5.3%)	1 (2.1%)	4 (8.5%)	
	>3 mm	35 (37.2%)	17 (36.2%)	18 (38.3%)	
Complication	Yes	2 (2.0%)	1 (1.0%)	1 (1.0%)	0.495
	No	92 (98.0%)	91 (99.0%)	91 (99.0%)	
Tumor progression	No	90 (95.7%)	45 (95.7%)	45 (95.7%)	1.000
	Yes	4 (4.3%)	2 (4.3%)	2 (4.3%)	
Cost (CNY)		12,799.27 ± 638.48	12,834.01 ± 665.784	12,762.99 ± 614.046	0.537
RFA power (Watt)		6.23 ± 2.416	6.06 ± 1.983	6.40 ± 2.795	0.739
RFA time (min)		5.16 ± 2.140	5.40 ± 2.335	4.92 ± 1.922	.450
RFA energy (KJ)		1.88 ± 0.904	1.88 ± 0.903	1.89 ± 0.914	0.736

RFA, radiofrequency ablation; CDFI, color Doppler flow imaging.

**Figure 2:**
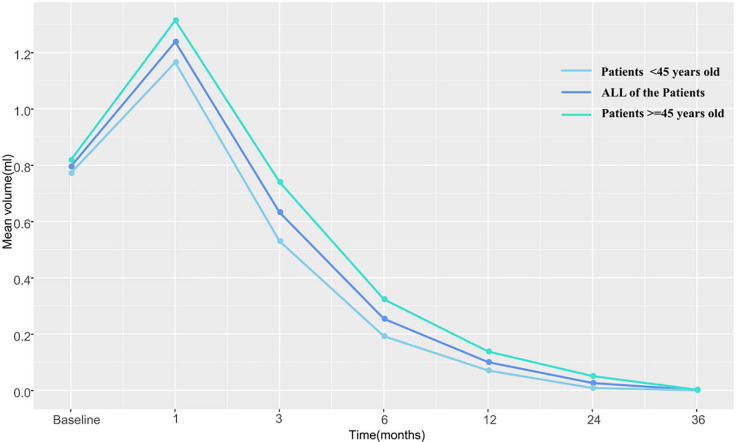
The changes in mean tumor volume at each follow-up point in both age subgroups.

The pre-RFA mean tumor volumes in patients <45 and in those ≧45 years of age were 0.772 and 0.818 cm^3^, respectively. These values were observed to decrease gradually during the follow-up period and became 0 and 0.002 cm^3^ in the former and latter subgroup, respectively, by the third postoperative year. The VRR according to age-subgrouping also changed positively from −65.8 and −86.6% at 1-month to 100.0 and 99.7% at the 36-month postprocedural follow-up, respectively. In the early and late stages after RFA, no differences were observed between both age subgroups. However, differences in volume and VRR were detected between the age subgroups at both the 3-month (P_V_=0.044 *vs* P_VRR_=0.031) and 6-month (P_V_=0.046 *vs* P_VRR_=0.039) follow-ups (**Table 3** and **Figures 2** **,** **3**).

**Table 3:** Changes in nodules volume and VRR in both age groups over the study period.

Time	Mean volume (cm^3^)				p	Mean VRR (%)				p
	<45		≧45			<45		≧45		
	Mean	Standard deviation	Mean	Standard deviation		Mean	Standard deviation	Mean	Standard deviation	
Baseline	0.772	0.423	0.818	0.535	0.910					
Immediately	2.642	1.647	3.112	1.664	0.102	3.769	2.236	4.706	3.498	0.228
1 month	1.165	0.668	1.314	0.697	0.349	−0.658	0.996	−0.866	1.100	0.384
3 months	0.530	0.386	0.740	0.526	0.044*	0.303	0.441	0.004	0.760	0.031*
6 months	0.192	0.203	0.323	0.325	0.046*	0.742	0.275	0.577	0.486	0.039*
12 months	0.070	0.107	0.138	0.186	0.057	0.908	0.136	0.817	0.246	0.057
24 months	0.008	0.016	0.051	0.111	0.236	0.991	0.016	0.943	0.113	0.364
36 months	0		0.002	0.006	0.071	1.00		0.997	0.008	0.071

VRR, volume reduction rate. *P < 0.05.

**Figure 3:**
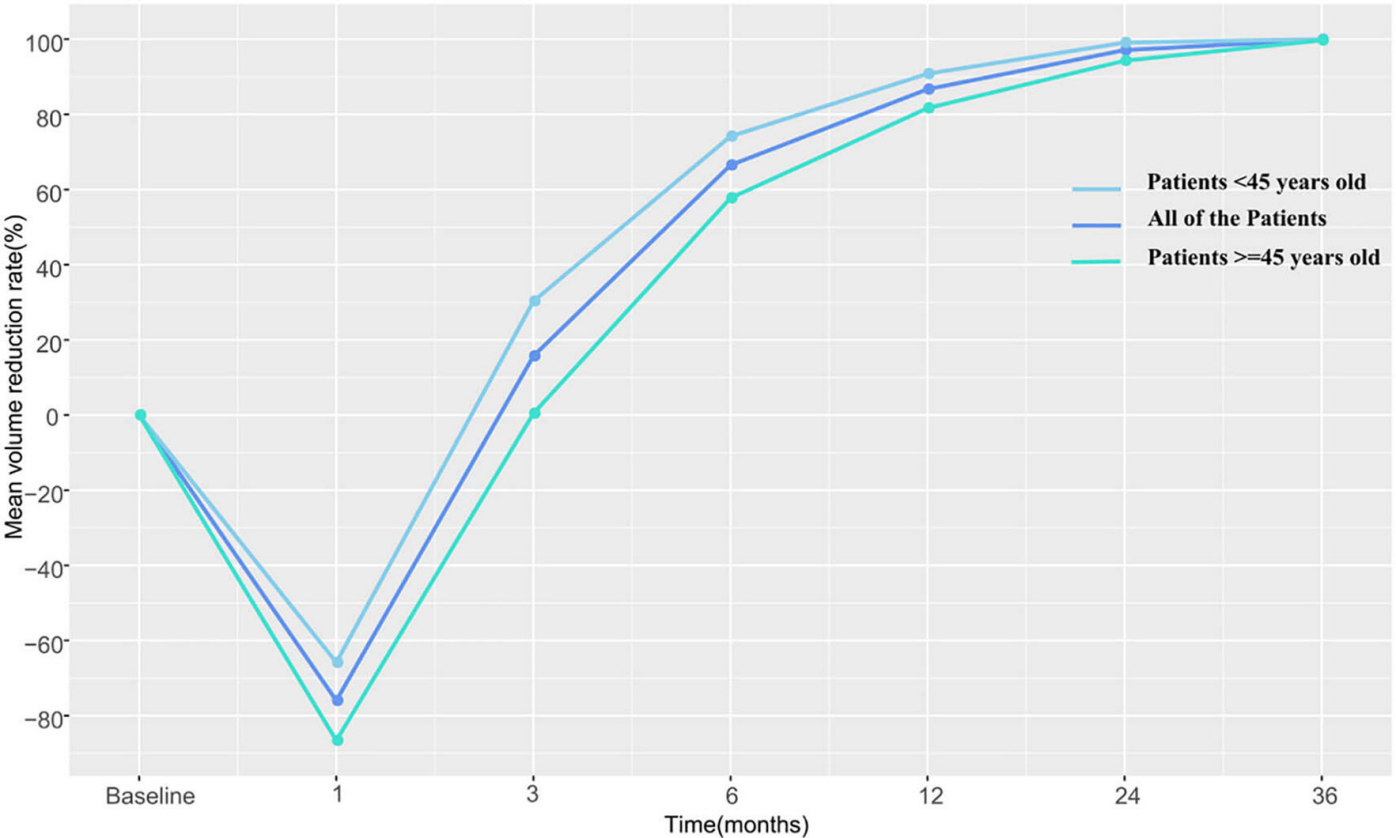
The changes in volume reduction rate at each follow-up point in both age subgroups.

### Outcomes of Surgical Resection According to Age Subgroups

Hospital costs (P=0.004) and operative duration (P=0.028) in the SR group differed significantly according to the age group of patients. Interestingly, as compared to younger patients, older patients required less operative time-interval but incurred higher costs. Complications (P=0.540) and tumor progression rates (P=0.483) did not differ between the age subgroups (**Table 4**). In the SR group, seven patients experienced hoarseness after surgery, of whom four also suffered from choking, while one experienced numbness for a few days postoperatively. Another older patient suffered dizziness and nausea but was released after few hours later with primary management. In total, 5 (4.5%) patients developed tumor progression, but no significant difference was observed between the defined age subgroups.

**Table 4:** Outcomes of surgical resection according to age subgroups.

SR		Total	<45	≧45	p
Sex	Female	93 (84.5%)	51 (86.4%)	42 (82.4%)	0.554
	Male	17 (15.5%)	8 (13.6%)	9 (13.6%)	
Aspect_ratio	<1	17 (15.5%)	10 (16.9%)	7 (13.7%)	0.641
	>1	93 (84.5%)	49 (83.1%)	44 (86.3%)	
Edge	Yes	5 (4.5%)	1 (1.7%)	4 (7.8%)	0.181
	No	105 (95.5%)	58 (98.3%)	47 (92.2%)	
CDFI	0	21 (19.1%)	12 (20.3%)	9 (17.6%)	0.797
	I	32 (29.1%)	19 (32.2%)	13 (25.5%)	
	II	32 (29.1%)	16 (27.1%)	16 (31.4%)	
	III	25 (22.7%)	12 (20.3%)	13 (25.5%)	
Calcification	No	18 (16.4%)	6 (10.2%)	12 (23.5%)	0.161
	<1 mm	82 (74.5%)	47 (79.7%)	35 (68.6%)	
	1 mm< & <2 mm	7 (6.4%)	5 (8.5%)	2 (3.9%)	
	>3 mm	3 (2.7%)	1 (1.7%)	2 (3.9%)	
Complication	Yes	11 (10%)	7 (11.9%%)	4 (7.8%)	0.540
	No	99 (90%)	52 (88.1%)	47 (92.2%)	
Tumor progression	No	105 (95.5%)	56 (94.9%)	49 (96.1%)	0.483
	Yes	5 (4.5%)	3 (5.1%)	2 (3.9%)	
Cost (CNY)		20,079.49 ± 6,470.82	18,967.62 ± 6,563.934	21,365.77 ± 6,176.946	0.004*
Operation time (min)		109.62 ± 42.232	116.90 ± 43.614	101.20 ± 39.328	0.028*

SR, surgical resection; CDFI, color Doppler flow imaging. *P < 0.05.

### Comparative Analysis of Treatment Outcomes Between the Age Subgroups

**Figure 4:**
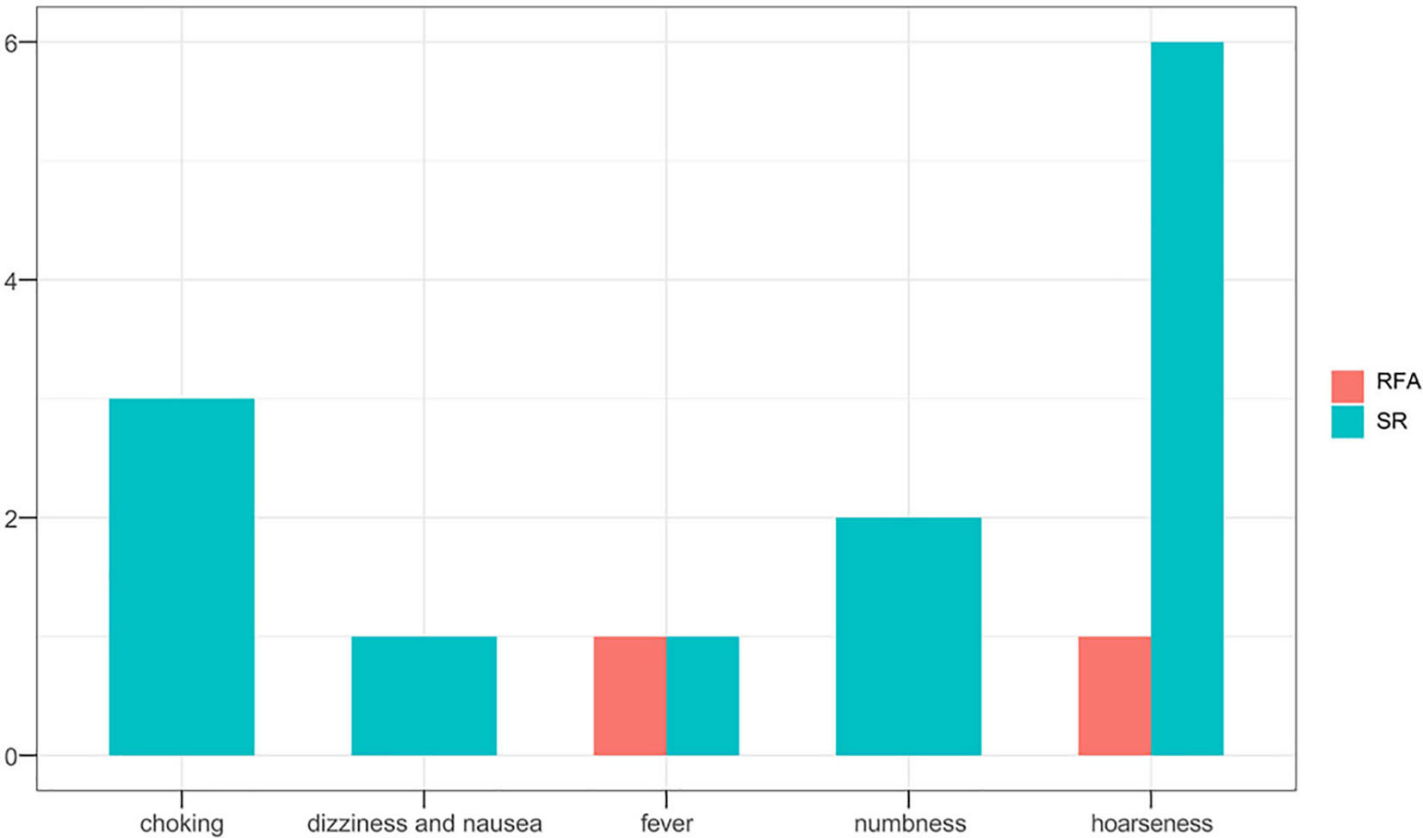
Complication recurrence in both treatment groups.

**Figure 5:**
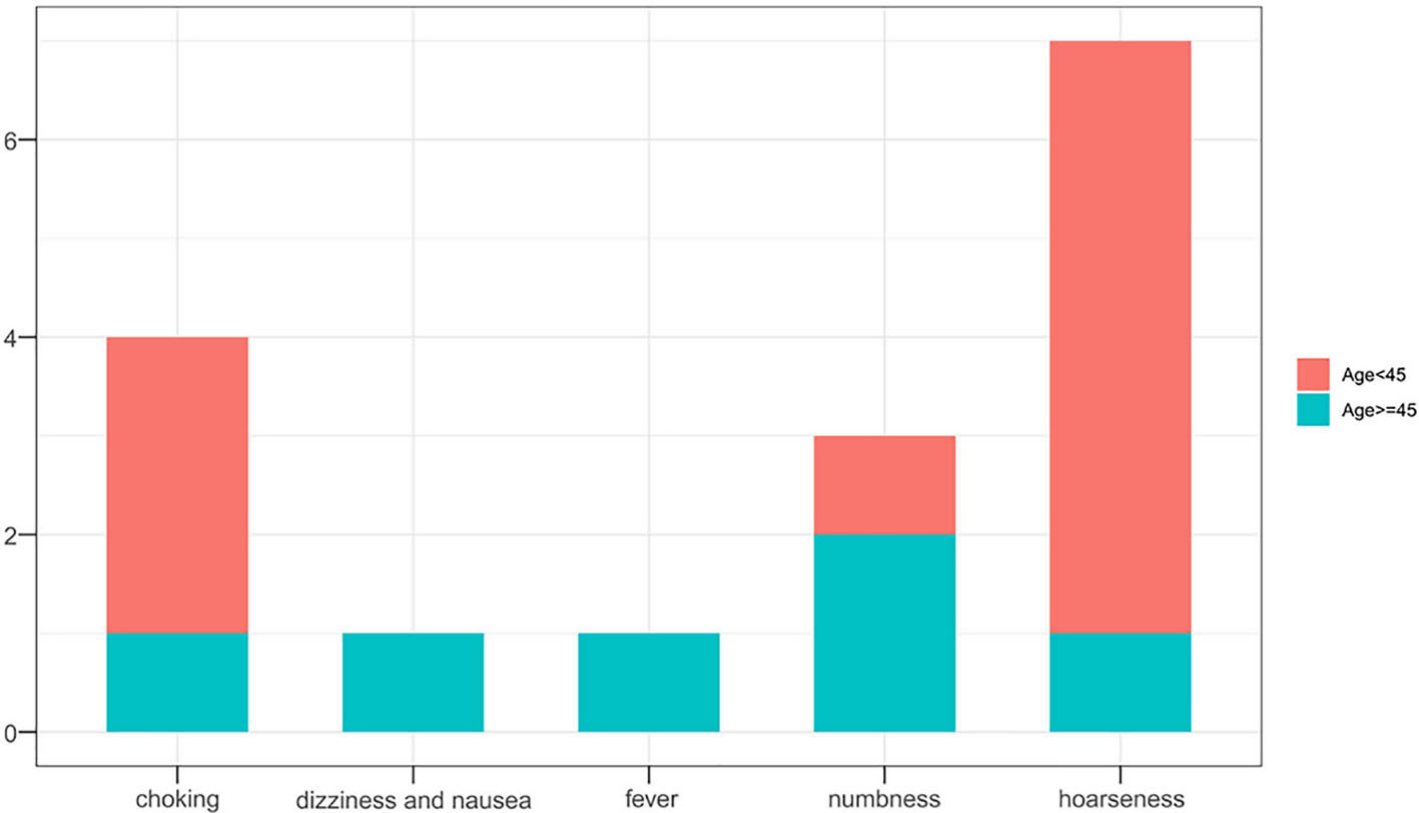
Complication recurrence following surgical resection in both age subgroups.

Overall outcomes of tumor progression and incidence of complications according to age subgroups in the two treatment groups are summarized in **Table 5**. No significant differences in tumor progression were observed between the subgroups (P=1.000). However, complications occurred more frequently in the SR group (P=0.023), especially in younger patients (P=0.015) (**Figures 4**, **5**). Moreover, significant differences in duration of procedure and hospital costs were observed between both younger and older patients from both treatment groups (P<0.001).

**Table 5:** Comparative analysis of treatment outcomes between the age subgroups.

	Total			<45			≧45			
	RFA	SR	P	RFA	SR	P	RFA	SR	P
Tumor progression	4	5	1.000	2	3	1.000	2	2	1.000
Complication	2	11	0.023*	1	7	0.015*	1	4	0.679
Procedure time (min)	5.16 ± 2.14	109.62 ± 42.232	<0.001*	5.40 ± 2.335	116.90 ± 43.614	<0.001*	4.92 ± 1.922	101.20 ± 39.328	<0.001*
Cost (CNY)	12,799.27 ± 638.478	20,079.49 ± 6,470.82	<0.001*	12,834.01 ± 665.784	18,967.62 ± 6,563.934	<0.001*	12,762.99 ± 614.046	21,365.77 ± 6,176.946	<0.001*

RFA, radiofrequency ablation; SR, surgical resection. *P < 0.05.

## Discussion

While thyroid surgery is still the first-line treatment option recommended for T1N0M0 PTC, according to both the 2015 ATA guideline and other guidelines for the management of thyroid cancer (4, 29, 30), clinical management of affected patients remains controversial due to indolent nature of the malignancy. Thyroidectomy was deemed an aggressive treatment option for low-risk PTC, especially in patients with stage I tumors. Moreover, the risk of complications including recurrent laryngeal nerve paralysis, hypoparathyroidism, and hypothyroidism not only significantly impacted the quality of life in affected patients but also made some patients unwilling to undergo SR.

Therefore, to prevent overtreatment, the ATA guidelines and those of the Japan Association mentioned that AS was safe in selected patients with low-risk PTC. According to the AJCC staging system, T1 PTC can be subdivided into T1a and T1b based on tumor diameter. AS in patients with T1aN0M0 was studied in the 1990s in Japan, and thereafter studies were conducted in other countries with huge populations, over long follow-up periods. However, research on T1bN0M0 stage PTC is still nascent, and reported findings vary greatly (31). Other non-surgical options excepting AS, such as ultrasound-guided RFA and microwave ablation (MWA), have been used for primary management of T1N0M0 PTC with expected outcomes (21, 22, 32, 33). However, a lot of research is still needed to explore the suitable population of RFA.

Age is a unique factor that negatively affects prognosis of patients with PTC. Many studies have considered 45 years of age as the cut-off point for staging dichotomization (34) for two reasons. First, the median age of patients at diagnosis in most studies is 45 years old (35). Second, some research results like Miyauchi et al. (36) reported that PTC patients aged ≥45 years experienced a tumor progression rate of <20% during AS, as compared to the >20% rate observed in their younger counterparts. In this study, we compared the efficacy and safety of RFA and SR in patients with T1bN0M0 PTC between different age subgroups (<45 years *vs* ≥45 years).

No significant differences in treatment outcomes were observed between the two age subgroups. The difference in VRR between that calculated at the 3- and 6-month follow-ups after RFA in patients ≥45 years of age was lower than estimated in younger patients. However, there was no difference in the rate of tumor shrinkage between the two subgroups either in the early period within 1 month or in the long-term (≥1 year). RFA is a traumatic procedure that causes coagulative necrosis in the central zone and leads to aseptic inflammation in the early postprocedural stage (16). Therefore, the reduction in the ablation zone is not initially obvious. Later, fragments of the necrotic tissue are gradually engulfed by infiltrating macrophages and other inflammatory cells until the ablated tissue completely disappears. Our results showed that this absorption process in young patients was faster than that in elderly patients, but both age subgroups experienced comparable level of therapeutic benefit by 1 year after the procedure, which means that the final effect of RFA in patients of different age subgroups was consistent. Cao et al. (21) reported two patients <45 years and one patients >45 years who underwent ablation therapy developed tumor progression, but without comparing whether there were differences between age subgroups. In our study, two patients in each age subgroup recurred tumor progression with no significant difference.

In the SR group, the difference was mainly reflected in the hospital cost. Results showed that older patients who underwent SR incurred higher hospital costs than faced by their younger counterparts. This was mainly because older patients require more postoperative care than younger patients. This conclusion was also confirmed by Sahli Z.T. et al. (37); they found advanced age was related to higher rates of extended length of hospital stay and surgery-related readmissions. In terms of the efficiency and safety, Joseph et al. (38) also reported an increased complication recurrence rate and reduced disease-free survival in a meta-analysis performed to investigate survival prognosis and complications in elderly patients with PTC, who underwent thyroidectomy. However, the rates for complications and tumor prognosis did not differ significantly between the age subgroups in the present study.

Adam et al. (34) showed that patient age was a prognostic factor for cancer-related death; no patient experienced PTC-related deaths in our study. RFA exhibited a number of obvious advantages as compared to SR in both age groups. First, tumor progression did not differ significantly between the RFA and SR groups, which indicated that patients suffered with T1bN0M0 PTC who underwent RFA could achieve almost the same therapeutic effect as SR regardless of whether they were older or younger than 45 years old. Second, irrespective of age group, complication rate, procedure duration, and hospital costs associated with RFA treatment were lower than those observed with SR. What’s more, less invasive and shorter hospital stay allows patients to receive treatment more easily (39).

This study has certain limitations. Firstly, given its retrospective nature, selection bias could not be excluded. The decision to perform either RFA or SR was taken according to clinical considerations and based on a patient’s willingness to undergo either procedure, but not through a randomized treatment allocation process. Secondly, all information was collated from a single center, and multicenter data are needed to verify our observations. Thirdly, considering the indolent nature of PTC, the follow-up duration of this study was insufficient.

In conclusion, ultrasound-guided RFA appears to be a feasible and safe treatment alternative for the management of T1bN0M0 PTC in both young and older patients. This minimally invasive technique allows patients to avoid the trauma associated with SR. However, larger studies conducted over longer periods are needed to verify our finding.

## Data Availability Statement

The original contributions presented in the study are included in the article/supplementary material. Further inquiries can be directed to the corresponding authors.

## Ethics Statement

This study was approved by the Ethics Committee of the Chinese People’s Liberation Army General Hospital.

## Author Contributions

HH contributed to design, acquisition, analysis of data, and presented this study. QS, RW, and JZ contributed to data collection and analysis. YZ took full responsibility for the study data and revising it critically. The final version of the manuscript for submission was approved by the YL. All authors contributed to the article and approved the submitted version. The scientific guarantor of this publication is YL.

## Funding

This study was supported by the National Natural Science Foundation of China (grant no. 81771834 and no. 81901746) and the Medical Big Data and AI R&D Project of Chinese PLA General Hospital (2019MBD-040).

## Conflict of Interest

The authors declare that the research was conducted in the absence of any commercial or financial relationships that could be construed as a potential conflict of interest.

## Publisher’s Note

All claims expressed in this article are solely those of the authors and do not necessarily represent those of their affiliated organizations, or those of the publisher, the editors and the reviewers. Any product that may be evaluated in this article, or claim that may be made by its manufacturer, is not guaranteed or endorsed by the publisher.

## Abbreviations

PTC, papillary thyroid carcinoma; AJCC, American Joint Committee on Cancer; TNM, tumor, lymph node, metastasis; ATA, American Thyroid Association; AS, active surveillance; SR, surgical resection; RFA, radiofrequency ablation; CEUS, contrast-enhanced ultrasonography; CNB, core needle biopsy; MWA, microwave ablation.
